# Survival outcome and mortality rate in patients with migraine: a population-based cohort study

**DOI:** 10.1186/s10194-018-0889-4

**Published:** 2018-07-25

**Authors:** Tomor Harnod, Cheng-Li Lin, Chia-Hung Kao

**Affiliations:** 1 Department of Neurosurgery, Hualien Tzu Chi General Hospital, Buddhist Tzu Chi Medical Foundation, Hualien, Taiwan; 2 College of Medicine, Tzu Chi University, Hualien, Taiwan; 3 Management Office for Health Data, China Medical University Hospital, Taichung, Taiwan; 4 College of Medicine, China Medical University, Taichung, Taiwan; 5 Graduate Institute of Biomedical Sciences and School of Medicine, College of Medicine, China Medical University, No. 2, Yuh-Der Road, Taichung, 404 Taiwan; 6 Department of Nuclear Medicine and PET Center, China Medical University Hospital, Taichung, Taiwan; 7 Department of Bioinformatics and Medical Engineering, Asia University, Taichung, Taiwan

**Keywords:** Cohort study, Migraine, Mortality, National Health Insurance

## Abstract

**Background:**

Whether the patients with migraine have an elevated mortality risk in Taiwan is unclear.

**Methods:**

We analyzed a subset of the National Health Insurance Research Database of Taiwan and enrolled patients (≥20 years old) who received a diagnosis of migraine between 2000 and 2012. The migraine cohort was further divided into the ones ever with status migrainosus (SM) and non-status migraine (NM) subcohort and compared with a 1:4 age-, sex-, comorbidity-, and index date-matched comparison cohort. We calculated the adjusted hazard ratios (aHRs) and 95% confidence intervals (CIs) for subsequent mortality risk after adjustment for age, sex, and comorbidities.

**Results:**

Compared with the comparison cohort, the corresponding aHRs for mortality were 0.81 (95% CI = 0.76–0.87), 0.89 (95% CI = 0.80–0.98), and 0.78 (95% CI = 0.72–0.84) in the total migraine, SM, and NM cohorts, respectively. SM, male sex, comorbid alcohol-related illness, depression, and mental disorders were identified as risk factors for subsequent mortality. Comorbid alcohol-related illness significantly increased the mortality risk in patients with migraine.

**Conclusion:**

Taiwanese patients with migraine require comprehensive and universal medical care. These patients would benefit from controlling their migraines and reducing the subsequent mortality.

## Background

Migraine is a major disabling disease worldwide. Numerous epidemiological studies conducted in developed Western countries have found that migraines affect approximately 20% of the general population [1, 2]. An international study in developing Asian countries showed that migraine accounts for 66.6% (range: 50.9%–85.8%) of all headache services at neurological clinics [3]. Globally, migraine is approximately 2 times more prevalent in women than in men, particularly in young and middle-aged women during menstruation [2, 4, 5]. Migraine not only affects the central nervous system and disrupts daily life but also increases life-threatening comorbid psychological and cardiovascular diseases in patients [6, 7]. The prevalence for migraine has been investigated a lot for the past 50 years and described the burden of migraine on individuals and communities worldwide [5, 8]. Researchers have proposed that patients with migraine have a higher risk of mortality due to a high vulnerability to other fatal diseases. Although numerous studies have examined the markers for the long-term outcomes of migraine, the actual mortality risk in patients with migraine has remained unclear so far [5, 8, 9]. Therefore, further investigation is required to understand the correlated mortality in patients with migraine to elucidate future treatment strategies for migraine.

Treatment-resistant migraine or status migrainosus (SM) poses a unique and difficult challenge to headache specialists. Patients with such a condition may experience considerably more disability than patients with a classic, non-status migraine (NM) and thus may require specific pharmacological treatment or adopt psychological strategies to reduce the burden of the disease [10]. There might be different mortality risks in these 2 groups of patients compared with the general population. We used a nationwide, population-based database to investigate the subsequent mortality rates in patients ever with SM and NM. The findings of this study might constructively inform the development and implementation of effective treatment strategies to reduce the migraine burden in Taiwan.

## Methods

### Data source

This population-based cohort study used the Longitudinal Health Insurance Database (LHID) provided by the National Health Research Institutes in Taiwan. Since March 1995, the Taiwanese government has managed a National Health Insurance (NHI) program that provides comprehensive universal health insurance to approximately 99.9% of the Taiwan population (more than 23 million individuals) [11, 12]. The details of the LHID and NHI program have been well documented [13, 14]. In this study, diagnoses were coded according to the International Classification of Diseases, Ninth Revision, Clinical Modification (ICD-9-CM). This study was approved by the Ethics Review Board of China Medical University in Taiwan (CMUH-104-REC2–115).

### Participants

**Fig. 1:**
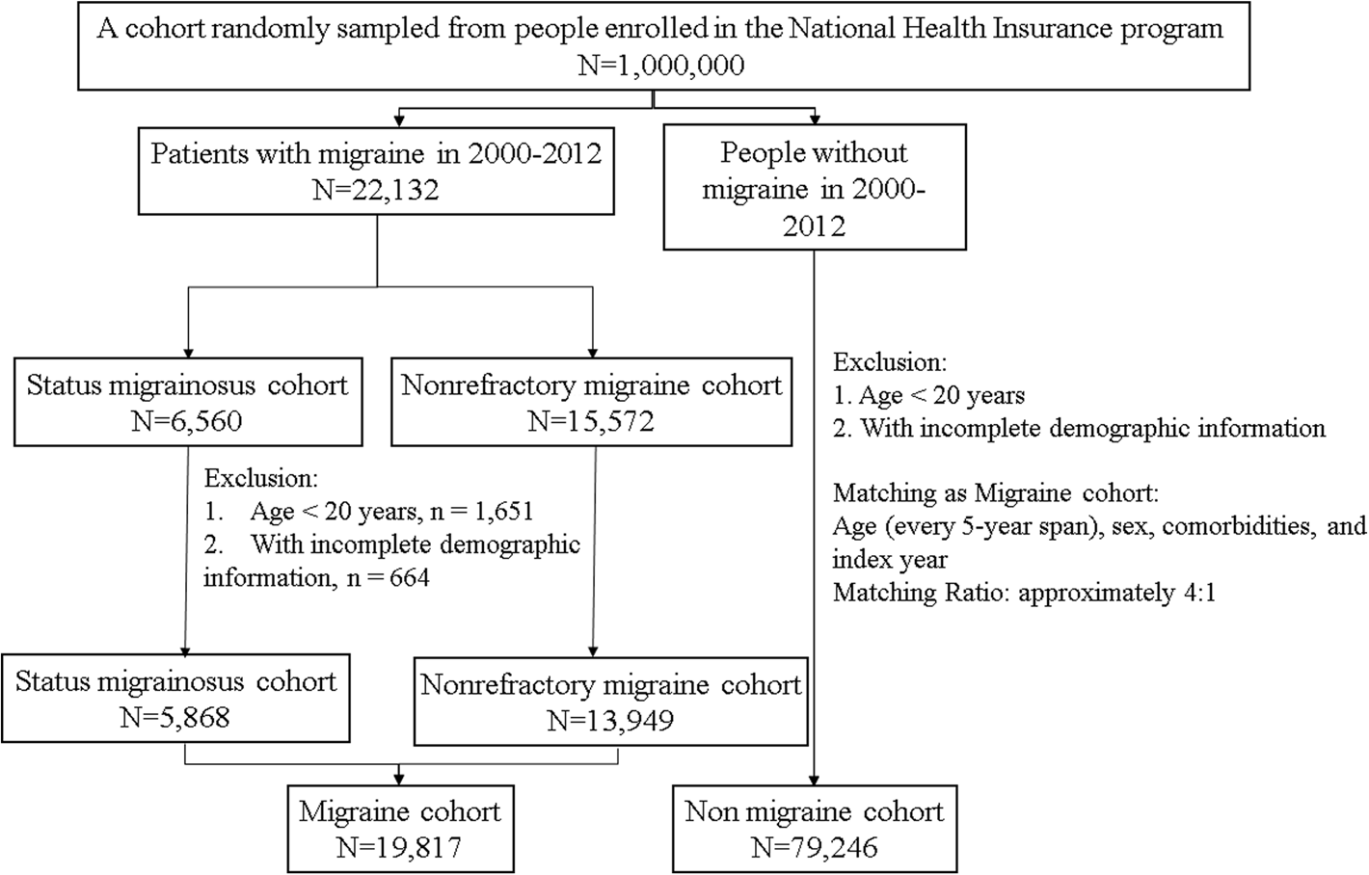
The flowchart of participant selection in the study cohorts

The migraine cohort was composed of patients with newly diagnosed SM (ICD-9-CM code 346.9, and without code 346.90 or 346.91) or NM (ICD-9-CM code 346.x except 346.9) from January 1, 2000 to December 31, 2012. The date of the diagnosis was defined as the index date. The comparison cohort comprised patients without a migraine diagnosis in the LHID. For each identified patient with migraine, 4 non-migraine participants from the LHID were randomly selected for the comparison cohort and were frequency matched with age (each 5-year span); sex; and comorbidities of depression (ICD-9-CM 296.2, 296.3, 296.82, 300.4, and 311), mental disorders (ICD-9-CM 290–319), insomnia (ICD-9-CM 307.4 and 780.5), alcohol-related illness (ICD-9-CM 291, 303, 305.00, 305.01, 305.02, 305.03, 571.0, 571.1, 571.3, 790.3, and V11.3), and anxiety (ICD-9-CM 300.00); and index date (year). Patients aged < 20 years or with incomplete demographic information were excluded (Fig. 1). The participants were followed until death, withdrawal from the NHI, or the end of 2013, whichever occurred first.

### Statistical analysis

The distributions of demographic characteristics, including age, sex, and comorbidities, were compared between the migraine and non-migraine cohorts. The differences were examined using the chi-square and *t* tests for categorical and continuous variables, respectively. We calculated the incidence density rates of subsequent mortality for the total migraine, SM, NM, and comparison cohorts. Univariable and multivariable Cox proportional hazard regression analyses were used to assess the risk of mortality associated with migraine compared with the comparison cohort. Hazard ratios (HRs) and 95% confidence intervals (CIs) were estimated in the Cox models. In a multivariable model, we adjusted for age, sex, and comorbidities of depression, mental disorders, insomnia, alcohol-related illness, and anxiety, all of which had a significant difference in the univariable model. A multivariable regression analysis was used to estimate the average number of hospital days per year for all-cause admissions and frequency of migraine-related medical visits annually, and those associated with the risk factors in patients with migraine were determined using a stepwise selection method. We used SAS (version 9.4, Statistical Analysis System Institute Inc., Cary, NC, USA) for the data analyses. A two-tailed *P* value of < 0.05 was considered indicative of statistical significance.

## Results

In total, 19,817 patients with migraine were selected (70.4% NM and 29.6% ever with SM). In both migraine and comparison cohorts, the mean age was 46 years (standard deviation [SD] = 15), proportion of women was 73.3%, and insomnia was the major comorbidity, followed by mental disorders and anxiety (Table 1).

**Table 1:** Distribution of age, sex, and comorbidities between the migraine and comparison cohorts

	Migraine						Comparison		*p*-value^b^
	Total *N* = 19,817		Status migrainosus *N* = 5868		Non-status migraine *N* = 13,949		*N* = 79,246		
	n	%	n	%	n	%	n	%	
Age, year									0.99
20–49	12,641	63.8	3685	62.8	8956	64.2	50,552	63.8	
50–64	4683	23.6	1388	23.7	3295	23.6	18,732	23.6	
65+	2493	12.6	795	13.6	1698	12.2	9962	12.6	
Mean (SD)^a^	45.9	14.9	46.3	15.3	45.7	14.8	45.8	15.2	0.37
Sex									0.99
Female	14,533	73.3	4199	71.6	10,334	74.1	5819	73.3	
Male	5284	26.7	1669	28.4	3615	25.9	21,127	26.7	
Comorbidity									
Depression	2536	12.8	663	11.3	1873	13.4	10,129	12.8	0.95
Mental disorders	10,928	55.1	3033	51.7	7895	56.6	43,690	55.1	0.97
Insomnia	13,414	67.7	3774	64.3	9640	69.1	53,645	67.7	0.99
Alcohol-related illness	746	3.76	199	3.39	547	3.92	2964	3.74	0.87
Anxiety	7316	36.9	2045	34.9	5271	37.8	29,248	36.9	0.98

Chi-square test; ^a^*t* test

^b^Total migraine vs. comparison

The overall incidence density rates of mortality were 9.74, 7.95, 9.02, and 7.49 per 1000 person-years in the comparison, total migraine, SM, and NM cohorts, respectively (Table 2). Compared with the comparison cohort, the corresponding adjusted HRs (aHRs; 95% CIs) of the mortality were 0.81 (0.76–0.87), 0.89 (0.80–0.98), and 0.78 (0.72–0.84) for the total migraine, SM, and NM cohorts, respectively. Patients aged 50–64 years (aHR = 2.57, 95% CI = 2.40–2.75) and ≥ 65 years (aHR = 10.5, 95% CI = 9.84–11.1) had a higher risk of mortality when compared with those aged 20–49 years. In the multivariable model, the mortality risk was higher in men (aHR = 1.78, 95% CI = 1.70–1.87) and in patients with comorbid depression (aHR = 1.33, 95% CI = 1.24–1.43), mental disorders (aHR = 1.19, 95% CI = 1.12–1.27), and alcohol-related illness (aHR = 2.18, 95% CI = 1.98–2.40). Furthermore, the NM cohort had a significantly lower mortality risk (aHR = 0.89, 95% CI = 0.79–0.99) than did the SM cohort (Table 2).

**Table 2:** Incidence and hazard ratios of mortality and associated risk factors

	HR (95% CI)					
	Event no	Person-years	Rate	Crude	Adjusted^a^	Adjusted^a^
Migraine						
None	5567	582,126	9.74	1.00	1.00	
Total	1167	146,776	7.95	0.82(0.77, 0.87)***	0.81(0.76, 0.87)***	
Status migrainosus	401	44,471	9.02	0.92(0.83, 1.02)	0.89(0.80, 0.98)*	1.00
Non-status migraine	766	102,304	7.49	0.77(0.72, 0.83)***	0.78(0.72, 0.84)***	0.89(0.79, 0.99)*
Age, year						
20–49	1753	475,258	3.69	1.00	1.00	1.00
50–64	1667	171,118	9.74	2.65(2.48, 2.84)***	2.57(2.40, 2.75)***	3.05(2.58, 3.61)***
65+	3414	82,525	41.4	11.4(10.8, 12.1)***	10.5(9.84, 11.1)***	12.4(10.6, 14.4)***
Sex						
Female	3693	538,264	6.86	1.00	1.00	1.00
Male	3141	190,637	16.5	2.41(2.29, 2.52)***	1.78(1.70, 1.87)***	1.96(1.74, 2.21)***
Comorbidity						
Depression						
No	5771	643,710	8.97	1.00	1.00	1.00
Yes	1063	85,192	12.5	1.41(1.32, 1.50)***	1.33(1.24, 1.43)***	1.27(1.07, 1.52)**
Mental disorders						
No	2279	320,327	7.11	1.00	1.00	1.00
Yes	4555	408,575	11.2	1.56(1.49, 1.64)***	1.19(1.12, 1.27)***	1.19(1.02, 1.39)*
Insomnia						
No	1985	263,064	7.55	1.00	1.00	1.00
Yes	4849	465,838	10.4	1.41(1.34, 1.48)***	0.95(0.89, 1.00)	1.05(0.92, 1.21)
Alcohol-related illness						
No	6367	708,326	8.99	1.00	1.00	1.00
Yes	467	20,575	22.7	2.61(2.37, 2.86)***	2.18(1.98, 2.40)***	1.95(1.53, 2.49)***
Anxiety						
No	3970	463,834	8.56	1.00	1.00	1.00
Yes	2864	265,067	10.8	1.27(1.21, 1.33)***	0.81(0.76, 0.87)***	0.79(0.67, 0.92)**

Rate per 1000 person-years

^a^: multivariable analysis of age, sex, and comorbidities of depression, mental disorders, insomnia, alcohol-related illness, and anxiety

*P < 0.05, ** *P* < 0.01, ***P < 0.001

Table 3 shows that the mortality risk, stratified by sex, age, and comorbidities; it was significantly lower in the total migraine and NM cohorts than in the comparison cohort. The aHRs of mortality were 0.68–0.86 and 0.64–0.84 in the total migraine and NM cohorts, respectively. In the total migraine cohort, patients with comorbidities demonstrated higher mortality rate than did those without. In patients with SM, the relative risk of mortality was lower in those aged 20–49 years (aHR = 0.79, 95% CI = 0.64–0.98) and in those without comorbidities (aHR = 0.73, 95% CI = 0.57–0.94; Table 3).

**Table 3:** Incidence and hazard ratios of mortality stratified by age, sex, and comorbidity for patients with migraine comparing with those without migraine

	Comparison *N* = 79,246		Migraine								
			Total *N* = 19,817			Status migrainosus *N* = 5868			Non-status migraine *N* = 13,949		
	Event no	Rate	Event no	Rate	HR^a^ (95% CI)	Event no	Rate	HR^a^ (95% CI)	Event no	Rate	HR^a^ (95% CI)
Age, year											
20–49	1495	3.94	258	2.70	0.68(0.60, 0.78)***	91	3.13	0.79(0.64,0.98)*	167	2.51	0.64(0.54, 0.75)***
50–64	1373	10.0	294	8.54	0.85(0.75, 0.96)**	86	8.53	0.85(0.68, 1.06)	208	8.55	0.85(0.73, 0.98)*
65+	2799	42.6	615	36.7	0.86(0.79, 0.94)***	224	42.0	0.97(0.85, 1.11)	391	34.2	0.81(0.73, 0.90)***
Sex											
Female	3090	7.19	603	5.57	0.78(0.71, 0.85)***	205	6.39	0.88(0.77, 1.02)	398	5.22	0.73(0.66, 0.81)***
Male	2577	16.9	564	14.7	0.86(0.78, 0.94)**	196	15.8	0.89(0.77, 1.03)	368	14.1	0.84(0.75, 0.94)**
Comorbidity											
None	986	7.56	172	5.20	0.69(0.59, 0.81)***	68	5.52	0.73(0.57, 0.94)*	104	5.02	0.66(0.54, 0.81)***
With any one	4681	10.4	995	8.75	0.84(0.79, 0.90)***	333	10.4	1.00(0.89, 1.11)	662	8.12	0.78(0.72, 0.85)***

Rate per 1000 person-years

^a^: multivariable analysis including age, sex, and comorbidities of depression, mental disorders, insomnia, alcohol-related illness, and anxiety

*P < 0.05, **P < 0.01, ***P < 0.001

Table 4 shows the stepwise regression analysis for evaluating the factors associated with the average all-cause hospital days annually among the patients with migraine. Compared with the comparison cohort, the average hospitalization duration was 0.66 days longer for the patients ever with SM. Furthermore, alcohol-related illness, male sex, depression, mental disorders, and age increased the hospitalization duration by 4.10, 1.98, 1.08, 0.81, and 0.11 days in the patients with migraine (Table 4). Table 5 shows the stepwise regression analysis used to evaluate the factors associated with the frequency of medical visits annually in the patients with migraine. We found that only age was a mild positive predictor (0.04 higher frequency of medical visits) in patients with migraine (Table 5).

**Table 4:** Stepwise regression analysis for average hospital days per year (all-cause admission) among patients with migraine

Variable	Parameter Estimate	Standard Error	95% CI
Intercept	−3.71	0.30	(−4.30, − 3.12)***
Status migrainosus	0.66	0.20	(0.27, 1.05)***
Age	0.11	0.01	(0.09, 0.12)***
Sex (male vs. female)	1.98	0.21	(1.57, 2.39)***
Alcohol-related illness	4.10	0.49	(3.15, 5.06)***
Mental disorders	0.81	0.20	(0.41, 1.21)***
Depression	1.08	0.29	(0.51, 1.65)***

***P < 0.001

**Table 5:** Stepwise regression analysis for frequency of migraine-related visits per year among patients with migraine

Variable	Parameter Estimate	Standard Error	95% CI
Intercept	−0.49	0.91	(−2.29, 1.30)
Age	0.04	0.02	(0.002, 0.08)*

*P < 0.05

## Discussion

In this study, Taiwanese patients with migraine demonstrated a lower all-cause subsequent mortality rate. Overall, 9.74 and 7.95 mortality events per 1000 person-years occurred in the comparison and total migraine cohorts. In the patients with NM, the protective effects against mortality might have been due to the frequent medical care and physical examinations, particularly for the female patients. Women usually tend to use more medical services and spend more on health care than men do, with regard to primary care, physical therapy, and other medical support [15, 16]. However, the life expectancy for women generally being higher than that for men and the higher mortality risk among men can be confounding factors [17]. In the SM cohort, fewer protective effects were observed and those were noted only in patients aged < 50 years; even the frequency of medical visits increased with age in these patients.

We observed some common trends between the increase of average all-cause hospitalization duration annually and higher mortality risk in the cohorts. In addition to the risk factors of age and the condition of SM, alcohol-related illness, male sex, depression, and mental disorders were major risk factors for all-cause hospitalization and mortality in patients with migraine. Among the comorbidities, alcohol-related illness was the strongest risk factor for hospital admission and mortality in patients with migraine. In general, a small amount of alcohol may not trigger migraine. Several recent studies could not conclusively show whether alcohol triggers migraines [18–20]. Differences in cultural and ethnic values and practices affect alcohol consumption, which increases complexity in studying the relationship between alcohol drinking and migraine. In this study, we considered alcohol-related illness a major comorbidity and risk factor; it should be due to that the patients with this comorbidity had already experienced toxic effects from alcohol consumption along with their migraine. Migraine alters peoples’ neuroendocrinal, neurophysiological, and neuropsychological conditions [5–8], and increases the risk of cardiovascular diseases. In addition to cardiovascular risk, this study reveals that alcohol-related illness is another risk factor to cause premature mortality in Taiwanese patients with migraine.

To our knowledge, this is the first study to show the long-term mortality rates in patients with migraine: patients with SM and NM demonstrated diverse survival outcomes. After the NHI was implemented in Taiwan, the annual mortality rate in general population significantly decreased by 5.83% from 1996 to 1999 [21]. In this study, we only analyzed the mortality risks in patients with migraine who died after being treated at inpatient facilities, excluding mortality cases outside a hospital. The NHI program has been over 20 years which is a universally compulsive insurance covering more than 99% of Taiwan’s population and it is operated by the government in Taiwan. NHI guarantees the residents of Taiwan equal access of medical service regardless of socioeconomic status, background, and critical problems existing or not [12, 22]. The Taiwan’s universal healthcare system showed very few disparities in accessing inpatient service and ultimate outcomes between different hospitals and areas in Taiwan [23, 24], and the proportion of migraineurs died outside a hospital should be very rare. However, the detail underlying mechanisms need to be studied in the future to explain why Taiwanese patients with migraine have a lower mortality risk depicted in this study. Furthermore, patients ever with SM, of the male sex, and with alcohol-related illness, depression, or mental disorders have fewer protective effects from premature mortality, and could be said to have a malignant course of migraine. We hope that our findings highlight a future strategy for providing proper medical care to patients with migraine.

Because of our study design, which included a nationwide, population-based sample with little risk of recall and selection bias, our findings should be considered convincing in Taiwan and other developing Asian countries with a heritage similar to that of Taiwan. However, this study has several limitations. First, we could not directly contact our patients because their identities were anonymized in the LHID. Therefore, the study design did not include details regarding the migraines, such as duration and frequency, psychological burden, and whether or how the migraines were treated with medication. Whether any migraine medications and other comorbid disorders influence the mortality rate is unknown. Second, any mortality cases occurring outside a hospital were out of the scope of our study. We intentionally designed our study in those died after being treated at inpatient facilities to achieve results with high validity, despite the slight possibility of introducing underestimation bias into the results. Third, we have to admit there was a possible bias that missed or underdiagnosed SM and NM. However, the NHIRD covers a highly representative sample of Taiwan’s general population because the reimbursement policy is universal and operated by a single-buyer, the government in Taiwan. All insurance claims should be scrutinized by medical reimbursement specialists and peer review according to the standard diagnosed criteria in the study. If these doctors or hospitals make wrong diagnoses or coding, they will be punished with a lot of penalties. Therefore, the diagnoses of SM and NM in this study were highly reliable and our results indicate that the sample size was sufficient to statistically demonstrate the mortality risk in patients with migraine in Taiwan.

## Conclusions

This study revealed that Taiwanese patients with migraine benefit from comprehensive and universal health care for not only controlling their migraines but also reducing subsequent mortality risk. Our findings provide vital information for clinicians and for assisting global efforts for understanding and treating migraine.

## Abbreviations

aHR: Adjusted hazard ratio
CI: Confidence interval
ICD-9-CM: International Classification of Diseases, Ninth Revision, Clinical Modification
NHIRD: National Health Insurance Research Database
